# Assessment of the genetic diversity of the alleles of gliadin-coding loci in common wheat (Triticum aestivum L.) collections in Kazakhstan and Russia

**DOI:** 10.18699/vjgb-24-31

**Published:** 2024-06

**Authors:** M.U. Utebayev, S.M. Dashkevich, O.O. Kradetskaya, I.V. Chilimova, N.A. Bome

**Affiliations:** A.I. Barayev Research and Production Centre of Grain Farming, Shortandy-1, Akmola Region, Kazakhstan; A.I. Barayev Research and Production Centre of Grain Farming, Shortandy-1, Akmola Region, Kazakhstan; A.I. Barayev Research and Production Centre of Grain Farming, Shortandy-1, Akmola Region, Kazakhstan; A.I. Barayev Research and Production Centre of Grain Farming, Shortandy-1, Akmola Region, Kazakhstan; University of Tyumen, Tyumen, Russia

**Keywords:** gliadin-coding loci, genetic diversity, genetic similarity, common wheat, electrophoresis, глиадинкодирующие локусы, генетическое разнообразие, генетическое сходство, мягкаяпшеница, электрофорез

## Abstract

The study of genetic resources using prolamin polymorphism in wheat cultivars from countries with different climatic conditions makes it possible to identify and trace the preference for the selection of the alleles of gliadine-coding loci characteristic of specific conditions. The aim of the study was to determine the “gliadin profile” of the collection of common wheat (Triticum aestivum L.) from breeding centers in Russia and Kazakhstan by studying the genetic diversity of allelic variants of gliadin-coding loci. Intrapopulation (μ ± Sμ) and genetic (H) diversity, the proportion of rare alleles (h ± Sh), identity criterion (I) and genetic similarity (r) of common wheat from eight breeding centers in Russia and Kazakhstan have been calculated. It has been ascertained that the samples of common wheat bred in Kostanay region (Karabalyk Agricultural Experimental Station, Kazakhstan) and Chelyabinsk region (Chelyabinsk Research Institute of Agriculture, Russia) had the highest intrapopulation diversity of gliadin alleles. The proportion of rare alleles (h) at Gli-B1 and Gli-D1 loci was the highest in the wheat cultivars bred by the Federal Center of Agriculture Research of the South-East Region (Saratov region, Russia), which is explained by a high frequency of occurrence of Gli-B1e (86 %) and Gli-D1a (89.9 %) alleles. Based on identity criterion (I), the studied samples of common wheat from different regions of Kazakhstan and Russia have differences in gliadin-coding loci. The highest value of I = 619.0 was found when comparing wheat samples originated from Kostanay and Saratov regions, and the lowest I = 114.4, for wheat cultivars from Tyumen and Chelyabinsk regions. Some region-specific gliadin alleles in wheat samples have been identified. A combination of Gli-A1f, Gli-B1e and Gli-Da alleles has been identified in the majority of wheat samples from Kazakhstan and Russia. Alleles (Gli-A1f, Gli-A1i, Gli-A1m, Gli-A1o, Gli-B1e, Gli-D1a, Gli-D1f, Gli-A2q, Gli-B2o, and Gli-D2a) turned out to be characteristic and were found with varying frequency in wheat cultivars in eight regions of Russia and Kazakhstan. The highest intravarietal polymorphism (51.1 %) was observed in wheat cultivars bred in Omsk region (Russia) and the lowest (16.6 %), in Pavlodar region (Kazakhstan). On the basis of the allele frequencies, a “gliadin profile” of wheat from various regions and breeding institutions of Russia and Kazakhstan was compiled, which can be used for the selection of parent pairs in the breeding process, the control of cultivars during reproduction, as well as for assessing varietal purity.

## Abstract (ru)

Изучение генетических ресурсов с использованием полиморфизма проламинов сортообразцов пшеницы из стран с различными климатическими условиями позволяет выявить и проследить предпочтительность отбора аллелей глиадинкодирующих локусов, характерных для конкретных условий. Цель исследования – определить «глиадиновый профиль» коллекции яровой мягкой пшеницы (Triticum aestivum L.) из селекционных центров России и Казахстана на основе изучения генетического разнообразия аллельных вариантов глиадинкодирующих локусов. Проведен расчет внутрипопуляционного (μ ± Sμ) и генетического (Н) разнообразия, доли редких аллелей (h ± Sh), критерия идентичности (I) и генетического сходства (r) яровой мягкой пшеницы из восьми селекционных центров России и Казахстана. Установлено, что наибольшим внутрипопуляционным разнообразием аллелей глиадина отличались образцы яровой мягкой пшеницы, созданные в Костанайской (Карабалыкская СХОС, Казахстан) и Челябинской (Челябинский НИИСХ, Россия) областях. Доля редких аллелей (h) по локусам Gli-В1 и Gli-D1 оказалась максимальной у сортов пшеницы селекции НИИСХ Юго-Востока (Саратовская область, Россия), что объясняется высокой частотой встречаемости аллелей Gli-В1е (86 %) и Gli-D1a (89.9 %). Статистически доказано, что изученные образцы яровой мягкой пше- ницы из разных областей Казахстана и России отличаются друг от друга по глиадинкодирующим локусам на основе критерия идентичности (I). Наибольшее значение I = 619.0 установлено при сравнении образцов пшеницы, происходящих из Костанайской и Саратовской областей, а минимальное I = 114.4 отмечено для сортов пшеницы из Тюменской и Челябинской областей. Выявлены аллели глиадина, которые были идентифицированы только образцах, созданных в определенных регионах. Сочетание аллелей Gli-А1f, Gli-B1e, Gli-Da идентифицировано у большинства образцов пшеницы Казахстана и России. Аллели Gli-A1f, Gli-A1i, Gli-A1m, Gli-A1o, Gli-B1e, Gli-D1a, Gli-D1f, Gli-A2q, Gli-B2o и Gli-D2a оказались характерными и с различной частотой встречались в сортах пшеницы восьми областей России и Казахстана. Наибольший внутрисортовой полиморфизм (51.1 %) наблюдался у сортов пшеницы селекции СибНИИСХ (Омская область, Россия), а наименьший (16.6 %) – у образцов Павлодарской СХОС (Павлодарская область, Казахстан). На основе частот встречаемости аллелей составлен «глиадиновый профиль» пшеницы из разных областей и селекционных учреждений России и Казахстана, который может быть использован для подбора родительских пар в селекционном процессе, контроле сортов при репродукции, а также для установления сортовой чистоты.

## Introduction

Over the decades, scientists have found that the use of electrophoresis of the wheat storage protein, gliadin, is one of the methods that make it possible to distinguish cultivars from each other (Autran et al., 1979; Watry et al., 2020). Differences in gliadin spectra are associated with the presence of allelic diversity of genes localized at the main loci: Gli-A1, Gli-B1, Gli-D1, Gli-A2, Gli-B2, Gli-D2. Locus alleles control the synthesis of several gliadin components, which are inherited linked together and form a block. At the same time, gliadin blocks may differ from each other in the number, intensity, electrophoretic mobility and molecular weight of the components (Sozinov, Poperelya, 1980).

Based on the study of the world wheat collection, allelic blocks of gliadin were identified and cataloged for common wheat (Metakovsky et al., 2018) and durum wheat (Melnikova et al., 2012). It has been established that the cultivars created in different breeding centers can be similar to each other in some alleles of gliadin-coding loci (Novoselskaya-Dragovich et al., 2011; Melnikova et al., 2012), despite the fact that no special allele selection was performed. The reason for this is probably the linkage of these alleles with genes or groups of genes that affect the selection-relevant traits of wheat (Xynias et al., 2006); it may also be due to the involvement in the breeding process of the same genotype (“masterpiece cultivar”), valuable for many biological and economic traits, such as: Saratovskaya 29, Bezostaya 1, Mironovskaya 808, etc. Therefore, frequently occurring gliadin alleles in the samples created for specific climatic conditions can be used in the identification of cultivars and as markers of valuable traits in the breeding process, such as grain qualities and resistance to abiotic factors (Sozinov, 1985).

The data obtained on the basis of the polymorphism of storage proteins may not be inferior in informativeness to DNA markers. An additional advantage of using such markers for plant breeding is inexpensive equipment and ease of analysis. The analysis of the prolamins composition is still used in the identification of crop cultivars, i. e. alfalfa (Kakaei, Ahmadian, 2021), millet (Ma et al., 2022) and rice (Kaur et al., 2023), in the study of genetic control of the synthesis of storage proteins in oats (Lyubimova et al., 2020). The method of electrophoresis of storage proteins is recommended for use in the identification of varietal material in the UPOV rules for barley (Barley, UPOV Code(s)…, 2018) and wheat (Wheat, UPOV Code(s)…, 2022). For the identification and registration of wheat samples created in the Russian Federation, a methodological guide for electrophoresis of storage proteins has been published (Laboratory Analysis…, 2013). The use of the electrophoresis method for the identification of wheat cultivars is prescribed in the state standard of the Republic of Kazakhstan (ST RK, 2018) and the Republic of Mali (MN- 01-03, 2001).

The results of the studies of wheat based on protein polymorphism can become the basis for a strategy for selecting genotypes with a certain combination of gliadin alleles. At the same time, the study of genetic resources based on the polymorphism of wheat prolamins from countries with different climatic conditions makes it possible to identify and trace the preference for selection and to establish the gliadin “profile” of the cultivar characteristic of specific conditions. In previous studies, the predominant or “leading” alleles of wheat prolamins characteristic of Northern Kazakhstan and Russia have been identified (Utebayev et al., 2016, 2019a, 2021, 2022). However, it is important to determine the gliadin spectrum of common wheat, which is characteristic of a specific breeding institution in Kazakhstan and Russia. Such information reflects the direction of breeding, the intensity of involvement of wheat genotypes from other breeding institutions, and the likelihood of “genetic erosion”.

In this regard, the aim of the study is to determine the characteristic “gliadin profile” of common wheat (Triticum aestivum L.) samples created in various breeding centers in Russia and Kazakhstan, based on the study and statistical calculation of the genetic diversity of allelic variants of gliadin- coding loci.

## Materials and methods

The object of study was 347 (177 Russian and 170 Kazakh) cultivars and breeding lines of common wheat (Supplementary Material 1)1, the gliadin spectra of which were described and published earlier (Dobrotvorskaya et al., 2009; Novoselskaya- Dragovich et al., 2013; Utebayev et al., 2016, 2019a, 2022).

Unfortunately, it was not possible to carry out a temporal periodization by years of creation for all cultivars and breeding lines. Therefore, the calculations were based on the principle of belonging of a particular sample to a breeding institution (region). The genetic formulas of gliadin from common wheat samples created in ten breeding institutions in Russia and Kazakhstan were analyzed (see Supplementary Material 1). In addition, the electrophoresis of gliadin of a new cultivar Tselinnaya Niva (Akmola region) was carried out, the formula of which was included in the total number of analyzed wheat samples. Gliadin spectra of wheat were obtained according to the method proposed by E.V. Metakovsky (Metakov- sky, Novoselskaya, 1991), gliadins were identified according to the catalog of alleles of gliadin-coding loci (Metakovsky, 1991).


Supplementary Materials are available in the online version of the paper: https://vavilov.elpub.ru/jour/manager/files/Suppl_Utebayev_Engl_28_3.pdf


Gliadin loci are designated according to the wheat gene catalog: Gli-A1, Gli-B1, Gli-D1, Gli-A2, Gli-B2 and Gli-D2 (McIntosh et al., 2003). Loci alleles were denoted by letters of the Latin alphabet in the following sequence, i. e. the genetic formula of gliadin of the cultivar Chinese Spring: Gli-A1a, Gli-B1a, Gli-D1a, Gli-A2a, Gli-B2a, Gli-D2a has an abbreviated notation: a, a, a, a, a, a; while the genetic formula of the cultivar Mironovskaya 808: Gli-A1f, Gli-B1b, Gli-D1g, Gli-A2n, Gli-B2m, Gli-D2e, in abbreviated form looks like: f, b, g, n, m, e.

Statistical analysis. Intrapopulation diversity (μ), which demonstrates the frequency of different genotypes, was calculated according to L.A. Zhivotovsky (1991):

**Formula. 1.**
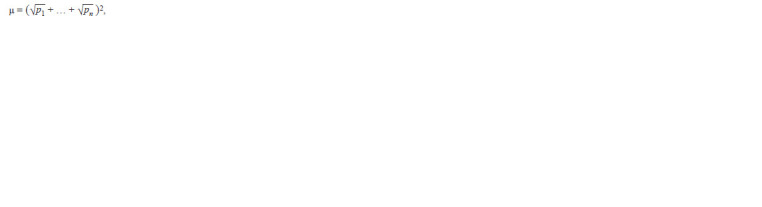
Formula1

where p is the frequency of alleles calculated by the formula: p = n/N, in which N is the sample size, n is the number of alleles of one locus in the cultivar (breeding line). With equal frequencies of all alleles of the locus μ = n, with an uneven distribution of frequencies μ < n, and with monomorphism μ = 1. The standard error of μ was calculated using the formula: Sμ = √μ(n – μ)/N ; where n is the number of alleles of one locus.

The calculation of the proportion of rare alleles (h) was determined by the formula:

**Formula. 2.**
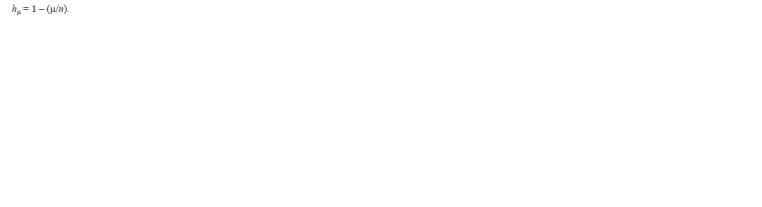
Formula2

To calculate the standard error of the proportion of rare alleles, the following formula was used:

**Formula. 3.**
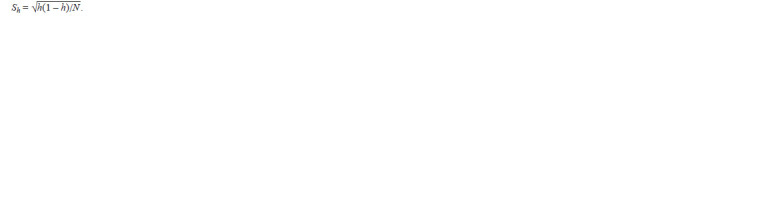
Formula3

In pairwise comparison of a group of wheat samples of different origins, the similarity index (r) was used (Zhivotovsky, 1979):

**Formula. 4.**
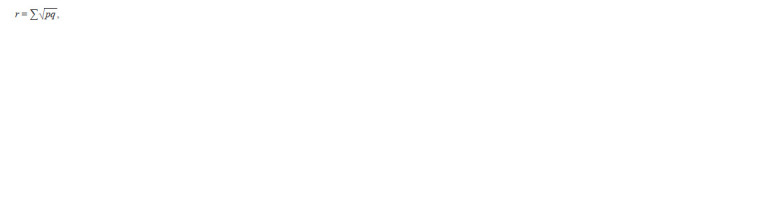
Formula4

where p is the frequency of the allele in the first population; q is the frequency of the allele in the second population. The statistical error of the r indicator was expressed by the formula:

**Formula. 5.**
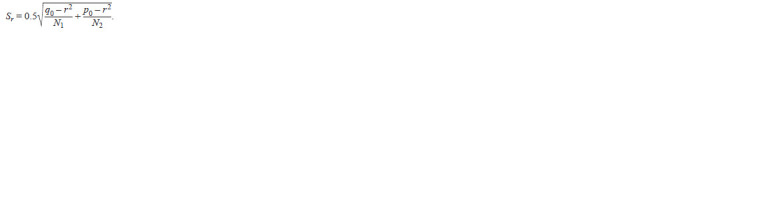
Formula5

In the case when all the identified alleles are common in the compared groups, the error was calculated using the formula

**Formula. 6.**
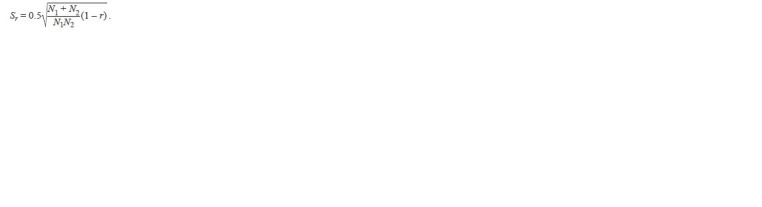
Formula6

Based on the similarity index (r), the identity criterion (I ) was calculated:

**Formula. 7.**
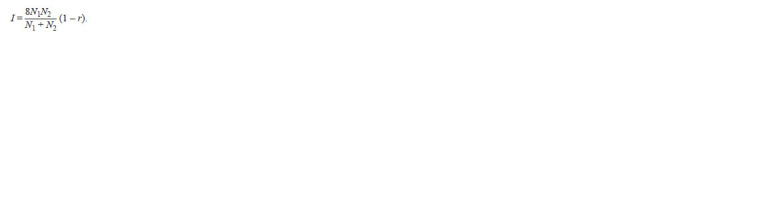
Formula7

At I exceeding the table value of χ2 with a 95 % significance level, cultivar populations were considered to have a significant difference.

The degree of genetic diversity (H ) is calculated according to M. Nei (1973):

**Formula. 8.**
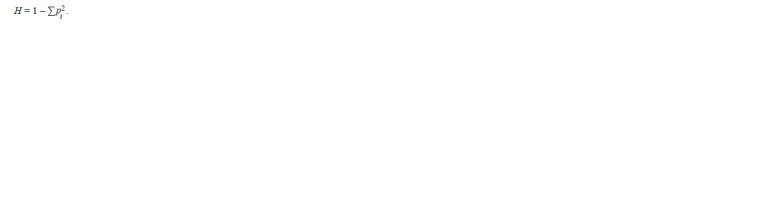
Formula8

## Results and discussion

The cultivars and breeding lines selected for study are presented in Supplementary Material 1. It is known that not all breeding lines reach the level of a cultivar, and not all cultivars reach the level of regionalization, nevertheless, this study presents the wheat samples (cultivars and breeding lines) that in one way or another used to be or are valuable for breeding, regardless of the year of creation or zoning. With this in mind, we made an attempt to show the allelic diversity of gliadincoding loci that is found in one or another breeding center in Russia and Kazakhstan.

*Gli-1* loci

**Fig. 1.**
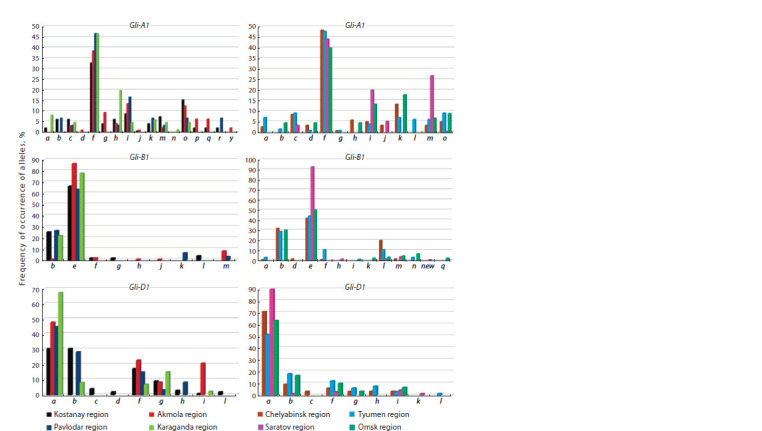
Frequency of occurrence of alleles (%) of Gli-1 loci of spring soft wheat by regions of Kazakhstan and Russia.

**Kazakhstan.** The number of identified alleles of A1 locus in wheat from Pavlodar and Karaganda regions was nine, from Akmola and Kostanay regions, 12 and 14 alleles, respectively (Fig. 1, Table 1). According to B1 locus, two alleles were identified in the wheat of Karaganda origin, four were from Pavlodar, five and six alleles were of Kostanay and Akmola regions, respectively.

**Table 1.**
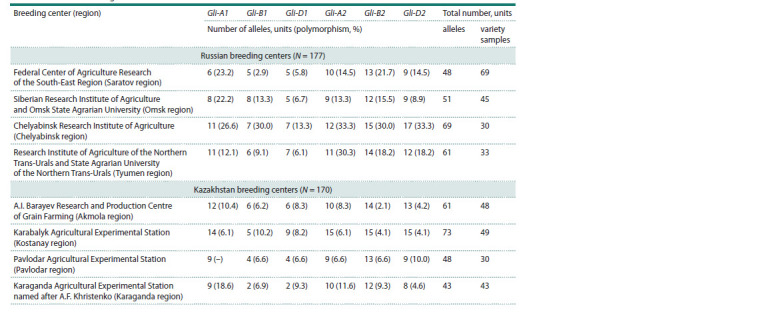
Number of alleles and polymorphism of Gli- loci in varieties of spring soft wheat created in various breeding centers in Russia and Kazakhstan

According to D1 locus, the greatest diversity was recorded in the wheat from Kostanay region (nine alleles), the minimum from Karaganda (two alleles), for Akmola and Pavlodar regions, six and four alleles were recorded, respectively. The analysis of the gliadin formulas showed that alleles found in wheat in one region were absent in another. Thus, Gli-A1d and Gli-A1y were identified only in Akmola wheat, and Gli-A1n in the sample from Karaganda region

At the same time, in the wheat samples from Akmola, Kostanay, Pavlodar and Karaganda regions, Gli-A1f, Gli-A1i and Gli-A1o alleles were distributed at Gli-A1 locus. Gli-A1f allele was common in all regions, its occurrence was 38.5 % in Akmola region, 32.9 % in Kostanay region, 46.7 % in Pavlodar region, and 46.5 % in Karaganda region (Fig. 1). The second most common allele in the wheat of Kostanay selection was Gli-A1o (15.3 %), and in Pavlodar, Gli-A1i with a frequency of 16.67 %. It should be noted that Gli-A1o and Gli-A1i alleles occur in the wheat from Akmola region with a frequency of 12.50 and 13.54 %, respectively

It should be added that Gli-A1h allele identified in the wheat samples from Karaganda region with a frequency of 19.8 % was not widespread in other regions of Kazakhstan. On the other hand, the blocks of gliadin components controlled by Gli-A1h and Gli-A1i alleles are quite similar in the number and electrophoretic mobility of the components, differing only in the mobility of one component in the γ zone (Metakovsky, 1991).

Since each gliadin locus is characterized by multiple allelisms, it is not uncommon to have a polymorphism of a cultivar or line. That is, polymorphic samples are a mixture of caryopses that differ in alleles of one or more gliadincoding loci. Gli-1 loci polymorphism was 27.9 % (12 out of 43 samples) for the samples from Karaganda region, 20.4 % (10 out of 49 samples) for those from Kostanay region, 18.7 % (9 out of 48 samples) and 13.3 % (4 out of 30 samples) for the wheat from Akmola and Pavlodar regions, respectively. It should be noted that the values given in Table 1 characterize the polymorphism of a single locus. In this regard, the largest polymorphism at A1 and D1 loci is from Karaganda region – 18.6 and 9.3 %, respectively. Note that the polymorphism at B1 locus is more often represented by the combination e+b, while A1 locus is more often represented by f allele in various combinations. The lowest allelic diversity of Gli-B1 locus was observed in the wheat samples from Karaganda and Pavlodar regions – two and four alleles, respectively. In all regions, the highest percentage of occurrence was recorded for Gli-B1e allele (Fig. 1).

Gli-B1l found only in the samples of Lutescens 71 and Liniya 19CHS (Karabalyk Agricultural Experimental Station, Kostanay region) is of interest, since this locus is a marker of wheat-rye translocation. The genes included in this translocation control the plant’s resistance to a number of fungal diseases, such as various types of rust (brown, stem, yellow) and powdery mildew (Kozub et al., 2012). However, the presence of this translocation turned out to reduce the technological characteristics of the grain, which ultimately affects the baking quality of wheat (Sozinov, 1985). On the other hand, the negative effects of wheat-rye translocation can be neutralized by the presence of “good” glutenin subunits such as 1Dx5+1Dy10, 1Bx7+1By9 and 1Bx7+1By8 (Sharma et al., 2018). It should be stated that the cultivar Lutescens 71 contains the components 1Dx5+1Dy10 and 1Bx7+1By9 in terms of the composition of high-molecular glutenin subunits (Utebayev et al., 2019b).

Gli-B1b allele is widely distributed among the studied samples, with the exception of the wheat from Akmola region. The low frequency of occurrence of this allele is probably due to the fact that most of the cultivars of A.I. Barayev Research and Production Centre of Grain Farming were created on the basis of the cultivars of Federal Center of Agriculture Research of the South-East Region (Saratov region), which are characterized by Gli-B1e allele (Novoselskaya-Dragovich et al., 2003).

The largest polymorphism at Gli-D1 locus was observed for the wheat from Karaganda region, 9.3 %, and was expressed by the combination of Gli-D1g+a.

Gli-D1a, Gli-D1f and Gli-D1g alleles are common in the wheat from all four regions of Kazakhstan (Fig. 1). At the same time, Gli-D1a had the maximum frequency of occurrence. It should be noted that Gli-D1a and Gli-D1f alleles control gliadin blocks that are very similar in the number and electrophoretic mobility of components, with the exception of the most mobile component located in the γ zone. There is an opinion that the less gliadin blocks differ in component composition, the closer they are to each other in terms of nucleotide composition (Chebotar et al., 2012). In this case, it can be assumed that the influence of such blocks on qualitative characteristics may be similar

**Russia.** The number of identified alleles of A1 locus in the wheat from Chelyabinsk and Tyumen regions was 11, from Saratov and Omsk regions, six and eight alleles, respectively. The largest number of identified alleles for B1 locus was observed in the wheat from Omsk region – eight, the smallest one was in Saratov wheat – five (Fig. 1). Seven alleles were identified for D1 locus in the wheat from Chelyabinsk and Tyumen regions, while five alleles were identified in the wheat from Saratov and Omsk regions

The analysis of gliadin formulas showed that for each locus there were alleles characteristic only for the samples from one region, i. e. Gli-B1h, Gli-B1new and Gli-D1k alleles were found only in the wheat of Saratov selection (Dobrotvorskaya et al., 2009), Gli-B1i, Gli-B1k and Gli-B1q – Omsk selection (Novoselskaya-Dragovich et al., 2013), Gli-A1l and Gli-D1l – Tyumen selection, and Gli-B1d – Chelyabinsk region (Fig. 1).

The largest polymorphism of Gli-1 loci was observed in the wheat of Chelyabinsk origin – 33.3 % (10 out of 30 samples), then in Omsk wheat – 31.1 % (14 out of 45 samples), in Saratov wheat – 26.1 % (18 out of 69 samples) and the smallest one, in Tyumen wheat – 18.2 % (6 out of 33 samples). It should be noted that such samples as Kukushka 12-6, Milturum 12013, Rossiyanka, Chelyabinskaya 17, Selivanovskiy Rusak and Omskaya 9 turned out to be polymorphic for all three Gli-1loci, with the largest number of alleles per locus found in the cultivar Chelyabinskaya 17 (see Supplementary Material 1). 

According to A1 locus, the high frequency of occurrence of Gli-A1f allele was recorded in the wheat from Tyumen region – 47.8 %, from Chelyabinsk region – 48.5 %, from Saratov region – 44.3 %, and from Omsk region – 40.0 % (Fig. 1). It should be stated that the allele is common among Australian (Metakovsky et al., 1990), Iranian (Salavati et al., 2008), Ukrainian (Kozub et al., 2009) selection, as well as in the cultivars from Western and Eastern Siberia (Nikolaev et al., 2009) and may be associated with some economically valuable traits of wheat.

Gli-A1i, Gli-A1m and Gli-A1o alleles were also “common” (Fig. 1). As it turned out, Gli-A1m and Gli-A1o alleles make up the “gliadin profile” of the wheat from Canada, Mexico, Scandinavian countries, Spain, and China (Metakovsky et al., 2018).

According to B1 locus, Gli-B1e allele “is in the lead” in the wheat of four regions, with different occurrence (Fig. 1). It should be added that Gli-B1e has a wide distribution area among the wheat cultivars of Kazakh and Russian selection (Novoselskaya-Dragovich et al., 2003; Nikolaev et al., 2009; Utebayev et al., 2019a). Also, according to B1 locus, the largest number of alleles occurring in a certain region was identified as following: Gli-B1d in Chelyabinsk region, Gli-B1h and Gli-B1new in Saratov region, Gli-B1i, Gli-B1k and Gli-B1q in Omsk region. During the analysis of genealogies, it was found that the wheat from Federal Center of Agriculture Research of the South-East Region (Saratov region), for which Gli-B1e allele is characteristic, was actively involved in breeding when creating the wheat cultivars of Tyumen and Chelyabinsk selection (GRIS, 2017). In their turn, most of the cultivars of Federal Center of Agriculture Research of the South-East Region (Saratov region), in one way or another, originate from two cultivar-populations: the genetic formula of Poltavka is Gli-A1o+f+c+j, Gli-B1e+m, Gli-D1a, Gli-A2q, Gli-B2o+s, Gli-D2e+a and for Selivanovskiy Rusak it is Gli-A1f+i+j**, Gli-B1e+new, Gli-D1a+i, Gli-A2j+q+s, Gli-B2o+q, Gli- D2e+s (Novoselskaya-Dragovich et al., 2003). Historically, most Kazakh cultivars are based on the cultivars from Saratov and Omsk regions, so it is quite expected that the gliadin profile of the wheat of the two countries is similar. Nevertheless, DNA diagnostics methods have proven the phylogenetic difference between Kazakh and Russian cultivars (Shavrukov et al., 2014). Gli-B1b allele was often found with the frequency of 32.0 % in the wheat from Chelyabinsk region, 28.8 %, from Tyumen region, and 30.0 %, from Omsk region. Since Gli- B1b is distributed from Scandinavian countries to Australia (Metakovsky et al., 2018), it is likely valuable for breeding.

At Gli-D1 locus, the highest occurrence was found for Gli-D1a allele (Fig. 1). In addition, alleles such as Gli-D1b, Gli-D1f and Gli-D1i are common to four regions of Russia (Saratov, Omsk, Chelyabinsk, Tyumen). It is worth paying attention to Gli-D1b allele, which is characteristic of the wheat from France, Mexico, Portugal, Bulgaria, Serbia (Metakovsky et al., 2018), Iran (Salavati et al., 2008) and England (Chernakov, Metakovsky, 1994). Based on the study of proteolysis of wheat prolamins, it is proposed to use Gli-D1b together with Gli-D1a as markers of adaptability in spring bread wheat (Upelniek et al., 2003).

*Gli-2* loci

**Fig. 2.**
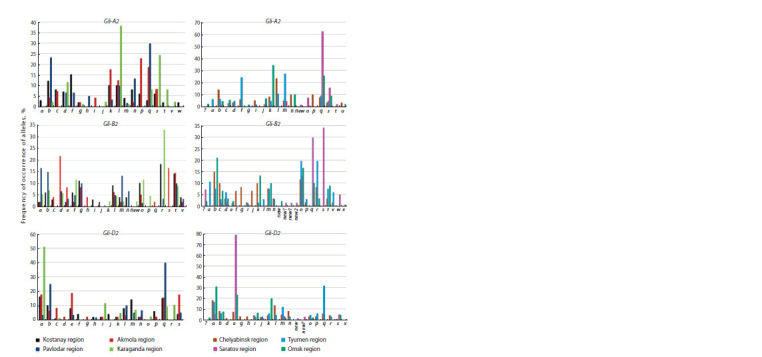
Frequency of occurrence of alleles (%) of Gli-2 loci of spring soft wheat by regions of Kazakhstan and Russia.

**Kazakhstan.** When analyzing the genetic formulas of gliadin at Gli-A2 locus, 10 alleles were identified in the wheat from Akmola and Karaganda regions. 15 and 9 alleles were identified in the wheat of Kostanay and Pavlodar selection, respectively. B2 locus is represented by 12 alleles in Karaganda wheat, 13 were found in Pavlodar wheat, 14, in Akmola wheat, and 15, in the wheat of Kostanay selection. According to D2 locus, eight alleles were identified in the wheat from Karaganda, nine, from Pavlodar, 13, from Akmola, and 15, from Kostanay regions (Table 1, Fig. 2).

Table 1 shows that the wheat of Karaganda selection is again “in the leading position” in terms of polymorphism of a single locus, since the values of A2 and B2 are the highest – 11.6 and 9.3 %, respectively. The “common” alleles were observed with varying frequencies in four regions of Kazakhstan: Gli- A2b, Gli-A2l, Gli-A2q, Gli-B2a, Gli-B2f, Gli-B2l, Gli-B2m, Gli-B2t, Gli-D2a and Gli-D2q.

The polymorphism of wheat by Gli-2 loci was 11.6 % (5 out of 43 samples) for Karaganda region, 10.4 % (5 out of 48 samples) for Akmola region, 10.0 % (3 out of 30 samples) and 8.2 % (4 out of 49 samples) for Pavlodar and Kostanay regions, respectively. Such specimens as Karabalykskaya 9 (Kostanay region), Lutescens 65, Lutescens 261 (Pavlodar region), Lutescens 1220, Lutescens 1242 (Karaganda region) turned out to be polymorphic at three Gli-2 loci.

The analysis of gliadin genetic formulas showed that the alleles Gli-A2v (2.3 %), Gli-B2k (2.3 %), Gli-B2new (2.3 %), Gli-B2p (4.6 %), Gli-D2o (2.3 %) and Gli-D2r (10.5 %) were found only in Karaganda wheat cultivars, and Gli-A2h (5.0 %), only in the samples of Pavlodar selection. Six alleles were identified in the samples from Kostanay region (Gli-A2a, Gli-A2w, Gli-B2i, Gli-B2j, Gli-D2f and Gli-B2j) and from Akmola region (Gli-B2h, Gli-B2q, Gli-B2s, Gli-D2d and Gli-B2g). At the same time, Gli-B2s allele with a frequency of 16.67 % is the second most common allele after Gli-B2d among the wheat of Akmola selection.

It should be stated that Gli-A2l allele, which occurs among Kazakh wheat samples, especially those from Karaganda region, turned out to be common among English (Chernakov, Metakovsky, 1994) and Iranian (Salavati et al., 2008) wheat samples. Also, Gli-A2f allele, which is the second most common wheat of Kostanay origin (15.31 %), was often found in Mexico and Portugal (Metakovsky et al., 2018). Gli-A2q allele, which has a high percentage of occurrence in Akmola and Pavlodar regions – 18.7 and 30.0 %, respectively, is of interest. It turned out that it is associated with the qualitative characteristics of grain, which are characteristic of strong cultivars of wheat (Dobrotvorskaya et al., 2009). On the other hand, it has been established that wheat genotypes with Gli- A2q allele have a long stem and low productivity (Khrunov et al., 2011).

Gli-B2s allele with a frequency of 16.7 %, identified only among the cultivars of Akmola region, constitutes the “profile” of the wheat of Saratov selection (Novoselskaya-Dragovich et al., 2003).

Gli-D2a allele, identified in the wheat samples from four regions of Kazakhstan, is widely distributed in common wheat cultivars from England (Chernakov, Metakovsky, 1994), Italy (Metakovsky et al., 1994), France (Metakovsky, Branlard, 1998), and Spain (Metakovsky et al., 2000). This is probably due to its association with adaptive traits, since the climate of European countries compared to Kazakhstan differs both in terms of precipitation, solar activity, and soil cover (Kunanbayev et al., 2022). Gli-D2q allele, which is found in the wheat from Pavlodar region, is widespread in Australia (Metakovsky et al., 1990), which may also be associated with economically valuable traits.

Russia. According to Gli-A2 locus, 12 alleles were identified in the wheat from Chelyabinsk region, 11, from Tyumen region, 10 and 9 alleles, from Saratov and Omsk regions, respectively. According to B2 locus, genetic diversity is represented by 12 alleles in the wheat from Omsk region, 13 al-leles, from Saratov region, 14 alleles, from Tyumen region, and 15 alleles, from Chelyabinsk region. According to D2 locus, 17 and 12 alleles were identified in the wheat from Chelyabinsk and Tyumen regions, respectively, whereas 9 alleles were found in the wheat of Saratov and Omsk selection (Table 1, Fig. 2).

Gli-A2b, Gli-A2k, Gli-A2q, Gli-A2s, Gli-B2c, Gli-B2d, Gli-B2o, Gli-D2a, Gli-D2m, and Gli-D2o alleles with different frequencies turned out to be “common” for the wheat samples from the analyzed areas. Polymorphism for all Gli-2 loci was stated for the wheat of Chelyabinsk origin at the level of 36.6 % (11 out of 30 samples), of Saratov origin – 34.8 % (24 out of 69 samples), of Omsk origin – 31.1 % (14 out of 45 samples) and of Tyumen origin – 30.3 % (10 out of 33 samples).

A high polymorphism of individual loci was observed for the wheat of Chelyabinsk origin: Gli-A2 (33.3 %), Gli-B2 (30.0 %) and Gli-D2 (33.3 %); the lowest one was found in the wheat of Omsk selection: Gli-A2 (13.3 %), Gli-B2 (15.5 %) and Gli-D2 (8.9 %) (Table 1).

Such samples as Kukushka 12-6, Milturum 12013, Rossiyanka, Uralskaya Kukushka, Chelyaba 2, Chelyabinskaya 17, Erythrospermum 24841 (Chelyabinsk region), Tyumenskaya 30, Surenta 4, Surenta 6, Rechka, Lutescens 70, Tyumenskaya Yubileynaya (Tyumen region), Lutescens 55- 11, Saratovskaya 50, Selivanovskiy Rusak (Saratov region), Pamyati Azieva (Omsk region) are polymorphic for three Gli-2 loci.

Based on the gliadin formulas, alleles that do not occur in other areas have been identified, i. e. eight alleles Gli-A2p, Gli-A2u, Gli-B2f, Gli-B2g, Gli-B2j, Gli-D2d, Gli-D2g and Gli-D2h have been identified only in the wheat of Chelyabinsk Research Institute of Agriculture; four alleles Gli-A2a, Gli- A2g, Gli-B2a and Gli-B2l, only in the cultivars from Tyumen region. 11 region-specific alleles Gli-A2o, Gli-A2t, Gli-B2s, Gli-B2w and Gli-B2x and several new alleles for each locus have been identified in the wheat from Saratov region (Dobrotvorskaya et al., 2009). Four alleles Gli-A2u and one new allele of A2, B2 and D2 loci were identified in the wheat of Omsk selection (Novoselskaya-Dragovich et al., 2013) (see Supplementary Material 1).

A high percentage of occurrence of Gli-A2q allele (62.5 %) was stated for the wheat of Federal Center of Agriculture Research of the South-East Region (Saratov region). At the same time, the highest allele frequency was identified for Gli-A2k (34.4 %) in the wheat of Omsk region and Gli-A2l (23.5 %) and Gli-A2m (27.3 %) for Chelyabinsk and Tyumen regions, respectively. It should be noted that Gli-A2l allele is common among the wheat from England (Chernakov, Metakovsky, 1994) and Iran (Salavati et al., 2008), and Gli-A2m allele, among the wheat from Canada and France (Metakovsky et al., 2018).

Gli-B2o allele turned out to be “common” for four regions of Russia. It has been stated that this allele is found in the wheat of Iranian (Salavati et al., 2008) and Italian (Metakovsky et al., 1994) origin, and in some cultivars of Saratov selection (Dobrotvorskaya et al., 2009), as well as in winter forms of wheat (Novoselskaya-Dragovich et al., 2015). In general, it should be added that according to B2 locus, the wheat of Saratov selection has the largest number of unknown alleles (Dobrotvorskaya et al., 2009).

In the cultivars of Tyumen origin, a high frequency of occurrence was stated for the following alleles: Gli-D2q (31.8 %) and Gli-D2a (16.6 %), respectively; whereas for the wheat of Chelyabinsk Research Institute of Agriculture, Gli- D2a (18.3 %) and Gli-D2l (13.7 %) alleles are predominant (Fig. 2). It should be noted that Gli-D2a allele is probably associated with valuable traits, since it is widely distributed among Italian wheat cultivars (Metakovsky et al., 2018), and among Omsk cultivars its occurrence reaches 31.1 % (Novoselskaya- Dragovich et al., 2013).

**Fig. 3.**
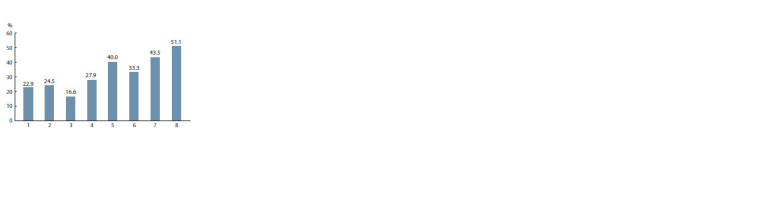
Polymorphism of spring soft wheat samples from various regions of Kazakhstan and Russia. 1 – Akmola region (A.I. Barayev Research and Production Centre of Grain Farming); 2 – Kostanay region (Karabalyk Agricultural Experimental Station); 3 – Pavlodar region (Pavlodar Agricultural Experimental Station); 4 – Karaganda region (Karaganda Agricultural Experimental Station named after A.F. Khristenko); 5 – Chelyabinsk region (Chelyabinsk Research Institute of Agriculture); 6 – Tyumen region (Research Institute of Agriculture of the Northern Trans- Urals and State Agrarian University of the Northern Trans-Urals); 7 – Saratov region (Federal Center of Agriculture Research of the South-East Region); 8 – Omsk region (Siberian Research Institute of Agriculture and Omsk State Agrarian University).

Thus, in eight regions of Russia and Kazakhstan, the following alleles have become widespread: Gli-A1f, Gli-A1i, Gli- A1m, Gli-A1o, Gli-B1e, Gli-D1a, Gli-D1f, Gli-A2q, Gli-B2o and Gli-D2a (Supplementary Material 2). In the analysis of the general polymorphism, heterogeneity in all six gliadin-coding loci was identified for four wheat samples of Chelyabinsk Research Institute of Agriculture (Kukushka 12-6, Milturum 12013, Rossiyanka, Chelyabinskaya 17) and one cultivar from Federal Center of Agriculture Research of the South-East Region (Selivanovskiy Rusak). Polymorphisms at five gliadin loci (A1, B1, A2, B2 and D2) were observed for the following samples: Karabalykskaya 9 (Karabalyk Agricultural Experimental Station), Lutesсens 1242 (Karaganda Agricultural Experimental Station named after A.F. Khristenko), Surenta 4 (Research Institute of Agriculture of the Northern Trans-Urals and State Agrarian University of the Northern Trans-Urals), Chelyaba 2 (Chelyabinsk Research Institute of Agriculture); heterogeneity of B1, D1, A2, B2 and D2 loci was stated for the cultivars Tyumenskaya 30 and Tyumenskaya Yubileynaya (Research Institute of Agriculture of the Northern Trans-Urals and State Agrarian University of the Northern Trans-Urals); the Lutesсens 55-11 sample (Federal Center of Agriculture Research of the South-East Region) is polymorphic at A1, D1, A2, B2 and D2 loci, and the Omskaya 9 sample (Siberian Research Institute of Agriculture and Omsk State Agrarian University) is polymorphic at A1, B1, D1, A2, and B2 loci (see Supplementary Material 1). The general polymorphism of the wheat samples depending on the origin is shown in Figure 3.

As can be seen, the greatest polymorphism was observed in the wheat of Omsk origin. It is believed that the presence of biotypes within the cultivar is an additional means to obtain a stable yield and increase its resistance to various environmental stress factors (Metakovsky et al., 2020).

Summarizing the results obtained by the frequencies of gliadin alleles, a “gliadin profile” of the wheat of Russian and Kazakh selection was compiled (Table 2). As can be seen, the combination of Gli-1 loci alleles (Gli-A1f, Gli-B1e and Gli-D1a) is the same for eight regions, while Gli-2 loci are different. The highest occurrence of the combination of Gli-A1f, Gli-B1e and Gli-D1a was in the samples of Saratov origin (33 out of 69 samples) and Karaganda origin (16 out of 43 samples) – 47.8 and 37.2 %, respectively; the lowest one was observed in the samples of Kostanay origin – 6.1 % (3 out of 49 samples). The maximum combination of Gli-A1f and Gli-B1e alleles found in the wheat from Akmola region (9 out of 48 samples) and Kostanay region (9 out of 49 samples) is 18.8 and 18.4 %, respectively, the minimum one is 1.4 % in the samples from Saratov region (1 out of 69 samples), and there are none from Tyumen region

**Table 2.**
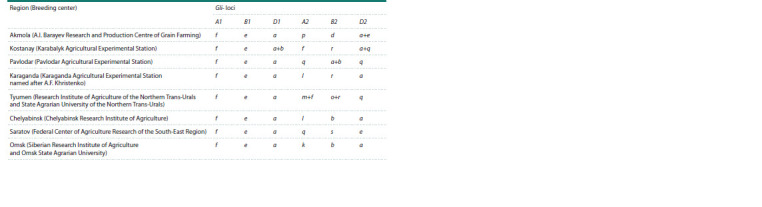
General “gliadin profile” of spring soft wheat created in various breeding centers from Russia and Kazakhstan

The association of Gli-B1e and Gli-D1a was most often found in the wheat from Saratov region (27 out of 69 samples) and Akmola region (13 out of 48 samples) – 39.1 and 27.1 %, respectively; only 9.1 % (3 out of 33 samples) are from Tyumen region. The information on the relationship between gliadin alleles and grain quality indicators is contradictory, i. e. the presence of Gli-A1m allele has been shown to cause a decrease in flour sedimentation. Later, it turned out that in most cases it is associated with Glu-A3e, the “worst” allele. On the other hand, Gli-A1m is present in many high-quality cultivars of Canadian selection (Metakovsky et al., 2019).

It was found that Gli-A2b and Gli-B2c alleles were statistically related to W-energy of the pastry deformation determined on the alveograph (Metakovsky et al., 1997). Although it has been suggested that alleles encoded by Gli-2 loci have a negative effect on grain quality (Masci et al., 2002), nonetheless, the use of Gli-A2s and Gli-B2o has been proposed as markers of increased protein, gluten content and grain nature (Khrunov et al., 2011). Later, on the basis of molecular genetic methods, the results were obtained indicating the presence of genes localized at Gli-2 loci that have a positive effect on the rheological properties of pastry (Noma et al., 2019).

Gli-B1e allele constitutes the “gliadin profile” of many high-quality wheat cultivars of Russian and Kazakh selection (Novoselskaya-Dragovich et al., 2013; Utebayev et al., 2019a, 2022), which is probably due to the fact that it encodes the synthesis of the so-called ω-gliadin d4, which is associated with increased grain quality (Branlard et al., 2003).

It is worth noting that not all gliadin alleles, which are “positioned” as markers of quality grain, increase qualitative characteristics. Weather and climatic conditions play a significant corrective role in the grain formation. Therefore, till the present moment, there is no information about “universal” alleles, the presence of which would contribute to the production of high-quality wheat grain. Such controversies concerning the relationship between gliadin alleles and grain characteristics contribute to an in-depth study of this phenomenon. On the other hand, the use of gliadin polymorphism for identification and determination of varietal purity does not lose its relevance due to the simplicity of execution and the constancy of the gliadin spectrum

Statistical analysis

Based on the statistical calculations, the intrapopulation (μ) and genetic diversity (H ) at A1, D1 and A2 loci turned out to be maximum for the wheat samples from Kostanay region; at B2 and D2 loci, for the samples from Chelyabinsk Research Institute of Agriculture, and at B1, for the cultivars from Tyumen region. The minimum values of μ and H were observed for the wheat from Akmola region at Gli-B1 – 2.78 ± 0.43 and 0.24, respectively (Supplementary Material 3).

It turned out that the applied H indicator cannot always describe the genetic diversity of the population satisfactorily, since it “underestimates” rare alleles (alleles with a low frequency of occurrence in the population or cultivar). Therefore, the additional application of the μ parameter allows for a more accurate assessment of the degree of diversity by taking into account the number of rare alleles and their frequency, i. e. 11 alleles were identified at Gli-A1 and Gli-A2 loci in the set of the cultivars of Tyumen origin. At the same time, the intrapopulation diversity μ for Gli-A1 locus was 8.00 ± 0.85, while for Gli-A2 it was 9.12 ± 0.72. This difference is explained by the fact that one allele with a frequency of 0.47 was “in the lead” for Gli-A1, and two alleles with frequencies of 0.24 and 0.27 prevailed for Gli-A2 locus. The applied indicator shows how variable the population is depending on the frequency of alleles

The rare allele ratio criterion (h) characterizes the distribution of frequencies, which is always h > 0 in case of unevenness, compared to μ, which evaluates the degree of diversity of the population. Based on this, the genetic and intrapopulation diversity at Gli-B1 and Gli-D1 loci turned out to be the lowest for the wheat samples from Saratov region (see Supplementary Material 3). Such a low value is explained by the predominance of Gli-B1e allele over other alleles

(92.8 % of occurrence). Accordingly, the h indicator will be the maximum – 0.56 ± 0.06. The same situation is observed in the analysis of allele frequencies at Gli-D1 locus. With a high percentage of occurrence of Gli-D1a allele (89.9 %), the value of parameters μ (2.46 ± 0.30) and H (0.19) decreases and the value of h increases accordingly. On average, the samples created in the Kostanay (10.15 ± 0.62) and Chelyabinsk (9.40 ± 0.76) regions had the highest intrapopulation diversity of alleles (Table 3). It should be noted that the intrapopulation diversity and the proportion of rare alleles in the wheat samples from Chelyabinsk region have increased markedly compared to the results that were published earlier: μ = 6.15 ± 0.33 and h = 0.12 ± 0.05 (Chernakov, Metakovsky, 1994). It should be noted that H values of the wheat bred in Tyumen are higher (0.78) than in Chelyabinsk wheat (0.77), but at the same time, the index of intrapopulation diversity (μ) in the wheat in Chelyabinsk region is higher.

**Table 3.**
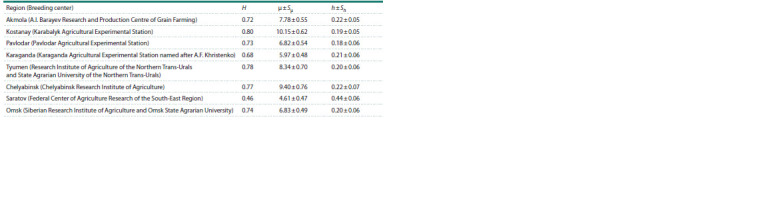
Average values of the proportion of rare alleles (h ± Sh), genetic (H ) and intrapopulation (μ ± Sμ) diversity at the Gli-1 and Gli-2 loci

If we take into account μ errors of both areas, then the difference in their values lies within the statistical error, and the intrapopulation diversity is approximately at the same level. However, the mean values were derived from calculations of the diversity of each locus, in which case the allelic diversity of cultivars (populations) within the locus must be taken into account. It turned out that with the same number (seven) of identified alleles of D1 locus, Gli-D1a allele prevailed in the wheat of Chelyabinsk Research Institute of Agriculture with a frequency of 71 %, and the rest had frequencies of no more than 10 %. At the same time, Gli-D1a allele was also “leading” in Tyumen cultivars, but with a lower frequency of 51.5 %, and Gli-D1b and Gli-D1f alleles with frequencies of 18.2 and 12.1 %, respectively, were found together with it. In other words, the diversity of Tyumen wheat cultivars at D1 locus is higher than that of Chelyabinsk wheat, which ultimately affected the average values of genetic and intrapopulation diversity.

Comparative analysis of genetic diversity of gliadin coding loci of common wheat in breeding centers of Kazakhstan and Russia

To determine the similarities and differences between the wheat samples from various breeding centers (regions) of Russia and Kazakhstan for gliadin alleles, a cluster analysis was carried out, as a result of which three groups A, B, and C were formed (Fig. 4). Group A consisted of the samples from North Kazakhstan regions, while the wheat of Kostanay and Pavlodar selection turned out to be quite close. This is explained by the fact that there were “common” alleles with different frequencies and for each locus, for example, out of 14 alleles identified by A1 locus, nine alleles turned out to be common, for a total of six loci out of 77 alleles, 45 were common, i. e. 58.4 %. Whereas in the wheat from Akmola region only 35.6 % of alleles were common to Kostanay and Pavlodar regions, which was reflected in the dendrogram. A similar situation was observed with the samples of cluster B. The “isolation” of Saratov wheat samples is due to the fact that only 10.1 % of gliadin alleles were common to the wheat from other regions of Kazakhstan and Russia.

For further establishment of the significant degree of differences between the groups of common wheat in terms of the frequency of occurrence of gliadin-coding loci alleles, the identity criterion (I ) was used. In its essence, if the obtained value exceeds the table value of χ2 at a given level of significance, then there is a significant difference between the groups (Zhivotovsky, 1979).

Supplementary Material 4 shows the values of genetic similarity (r), the criterion of pairwise similarity of the studied groups and the criterion of identity (I ) for each locus separately. Genetic similarity (r) does not exceed 1, but can be equal to 1 only if the groups being compared are identical in number and frequency of alleles. When averaged, the obtained values of the identity criterion (I ) exceeded the tabular value of χ2 for all pairwise comparisons. Accordingly, the studied groups of common wheat samples from different regions and breeding centers of Kazakhstan and Russia significantly differ from each other in gliadin-coding loci (Table 4).

**Table 4.**
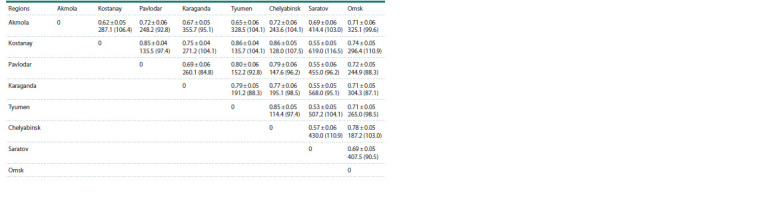
Average values of genetic similarity (r) and identity criterion (I ) of spring bread wheat samples for Gli-1 and Gli-2 loci by region of origin The upper number is the indicator of genetic similarity (r), the lower number is the criterion of identity (I ). In parentheses χ2 for a 5 % significance level, if I > χ2, then the differences are significant.

However, when analyzing the I values for individual loci, it turned out that even in the presence of alleles characteristic of a certain area, a significant difference between the groups was not always achieved (see Supplementary Material 4), i. e. when comparing the wheat of Tyumen and Omsk origin, the difference at D1 locus was insignificant I = 7.6 (12.6), since five out of seven identified alleles occurred in both groups, and with a fairly high frequency. Note that in most cases, there was a slight difference at Gli-1 loci, while at Gli-2 loci, the differences were statistically significant.

This is probably due to the fact that wheat breeding has traditionally been aimed at increasing yield, grain quality and resistance to various stressors, and alleles of Gli-A1, Gli-B1 and Gli-D1 loci are associated with baking quality (Nieto- Taladriz et al., 1994; Li et al., 2009; Demichelis et al., 2019), and resistance to leaf, stem rust (Czarnecki, Lukow, 1992; Cox et al., 1994) and powdery mildew (Hsam et al., 2015).

## Conclusion

Based on the study, description and statistical calculation of the genetic diversity of allelic variants of gliadin-coding loci of common wheat, a significant difference in genotypes from different regions of Kazakhstan and Russia has been established. The revealed genetic differentiation on the basis of protein polymorphism is likely adaptive. The gliadin alleles that are characteristic of a certain region have been identified. The “gliadin profile” of the wheat of Kazakhstan and Russian origin has been established, which shows the preference of wheat genotypes for gliadin alleles as a result of selection. This information can be used for the selection of parent pairs in the breeding process, the control of cultivars during reproduction, as well as for the establishment of varietal purity.

## Conflict of interest

The authors declare no conflict of interest.
